# Books and Magazines

**Published:** 1908-01

**Authors:** 


### Books and Magazines.

The Annual Output of Physicians' Visiting Lists is now on the market. We have received the 1908 edition of the popular favorite—Blakiston's—the standard for fifty-seven years. It needs no introduction or commendation. It is a beauty. It contains everything a doctor needs in the transactions of the business end of his calling. There is a perpetual edition (without dates), 1300 names, $1.25; 2600 names, $1.50, and a monthly edition (without dates), 75 cents and $1. Send for one.

---

The Cause and Prevention of Beriberi. By W. Leonard, M. B., B. S., F. R. C. S., State Surgeon, Negri, Sembilan, Federated Malay States. London: Rebman, Limited. New York: Rebman Company, 1907. Pp. xiii-544. Price, $6.

It has been well said that volumes could be written on beriberi. In fact, they have been. Dr. Braddon presents his subject in a logical manner, but his argument may be reduced in simple terms to an advocacy of the rice theory of the production of the disease. The first of the author's conclusions is as follows: "State, decorticated (white) rice, therefore, at times contains a poison, the effect of which is to produce beriberi." The author then continues: "Second, the agent which produces the poison in rice is specific of or peculiar to that grain. Third, the beriberi poison is not performed (or not present in quantities sufficient to cause symptoms) in normal, fresh rice seeds, but is adventitious. * * * Sixth, the poison of stale rice has an antecedent in fresh rice. The agent must be, therefore, some ferment or parasite or epiphyte peculiar to padi. Seventh, the beriberi poison is probably an alkaloid which is stable and not volatile, and resembles atropine and muscarine in some of its effects. Eighth, the formation of poison in stale rice is probably due neither to fermentation nor to bacteria, but to the growth in it of a special fungus. Ninth, the beriberi producing fungus of rice is probably a surface parasite or epiphyte affecting the seed saprophytically after decortication." The author then goes on to compare this fungus with that of toxic rye and lolium.

As is well known, students of and writers about beriberi are divided into two groups according to their advocacy of theories of etiology. One group, to which the author of the work under review belongs, believes that the disease is due to mouldy rice. The other group believes that the disease is due to a bacterium. Within the past year we have noticed in our editorial columns from time to time the various opinions of the nature of the micro-organisms. Further, an editorial in our issue of October 26 calls attention to the theory, advanced by Hewlett and de Korte, of the protozoan nature of the cause of the disease. It is needless to repeat that no one of the theories advanced so far has been proved. This much is in favor of the mouldy rice theory: Change of diet and removal of the patient to a non-beriberic place is of great assistance in curing the disease; at any rate, after the subsidence of the acute stage.

Dr. Braddon's book is an admirable piece of work and reveals the author's great industry. The literature has been exhaustively reviewed, and the references are given in all cases. From the mechanical point of view the book shows great merit; in spite of its size it is quite light and can easily be held in the hand.—*Ex.*

---

THE PRACTITIONER'S VISITING LIST FOR 1908.—An invaluable pocket-size book containing memoranda and data important for every physician, and ruled blanks for recording every detail of practice. The Weekly, Monthly and 30-Patient Perpetual contain 32 pages of data and 160 pages of classified blanks. The 60-Patient Perpetual consists of 256 pages of blanks alone. Each in one wallet-shaped book, bound in flexible leather, with flap and pocket, pencil and rubber, and calendar for two years. Price by mail, postpaid, to any address, $1.25. Thumb-letter index, 25 cents extra. Descriptive circular showing the several styles sent on request. Lea Brothers & Co., Publishers, Philadelphia and New York.

The text portion of the Practitioners' Visiting List for 1908 has been thoroughly revised and brought up to date. It contains among other valuable information a scheme of dentition; tables of weights and measures and comparative scales; instructions for examining the urine; diagnostic table of eruptive fevers; incompatibles, poisons and antidotes; directions for effecting artificial respiration; extensive table of doses; an alphabetical table of diseases and their remedies, and directions for ligation of arteries. The record portion contains ruled blanks of various kinds, adapted for noting all details of practice and professional business.

Printed on fine, tough paper suitable for either pen or pencil, and bound with the utmost strength in handsome grained leather, the Practitioners' Visiting List is sold at the lowest price compatible with perfection in every detail.

---

APPLETON'S MODERN CLINICAL MEDICINE.—Diseases of the Nervous System. Volume 4. Price, $7, cloth.

This splendid book is the fourth volume of Appleton's Modern Clinical Medicine, the masterpiece of the twentieth century; translated from *Die Deutsche Klinik* by the leading men in the German medical profession.

Volumes 1, 2 and 3 have recently been reviewed in this JOURNAL; i e., (1) Infectious Diseases, edited by Dr. Jas. C. Wilson, Philadelphia; (2) Diseases of Metabolism and of the Blood, Animal Parasites, Toxicology, edited by Dr. Richard C. Cabot, Boston; (3) Diseases of the Digestive System, edited by Dr. Frank Billings, Chicago. We have now volume 4, Diseases of the Nervous System, edited by Dr. Archibald Church, Professor of

Nervous and Mental Diseases in the Northwestern University, Chicago. This work has received a most favorable reception everywhere. Leading clinicians say that these books are five years in advance of the times.

---

KIRKE'S HAND-BOOK OF PHYSIOLOGY.—Sixth American Revision, Revised and Rewritten by Chas. Wilson Greene, A. M., Ph. D., Professor of Physiology and Pharmacology, University of Missouri. Seven hundred pages. Five hundred and seven illustrations, including many in colors. New York: William Wood & Co., 1907. Price, not stated.

Kirke's Hand-Book of Physiology is exceedingly popular, both with practicing physicians and students, for while it is sufficiently advanced and technical to meet all requirements of the former it is also sufficiently elementary for the beginner. A mistake made by many writers of text-books is in assuming too much knowledge of the subject on the part of the reader. Many beginners are like the man from Missouri; they have to be told some very elementary things. The text has been largely rewritten and much anatomical discussion omitted. Its place has been taken by new facts learned in the laboratory—bringing the subject up to the present state of knowledge on that branch of science. An entirely new feature is a comprehensive guide to or directions for laboratory work, one that will be especially helpful. Also, in the front is a table of measurements, giving the English equivalents of the French scale, and the Fahrenheit and centigrade scales side by side. Great importance attaches to the results of researches on the functions of the ductless glands in late years of Schafer and Oliver, Abel and others, and their discoveries, together with those of Sajous, throw much light upon the rôle played by the secretions of the thyroid and of the adrenals as affecting nutrition and metabolism.

---

Modern Medicine—Its Theory and Practice.—In Original Contributions by American and Foreign Authors. Edited by William Osler, M. D., Regius Professor of Medicine in Oxford University, England; formerly Professor of Medicine in Johns Hopkins University, Baltimore; in the University of Pennsylvania, Philadelphia and in McGill University, Montreal. Assisted by Thomas McCrea, M. D., Associate Professor of Medicine and Clinical Therapeutics in Johns Hopkins University, Baltimore. In seven octavo volumes of about 900 pages each, illustrated. Volume III, just ready. Price per volume: Cloth, $6, net; leather, $7, net; half Morocco, $7.50, net. Lea Brothers & Co., Publishers, Philadelphia and New York, 1907.

The appearance of the third volume of Osler's Modern Medicine marks the steady progress of this great work towards completion. In this volume the grand division of Infectious Diseases is concluded, and space is found for equally full consideration of Diseases of the Respiratory Tract. The fourth volume, now going through press, will cover Diseases of the Circulatory System and Blood. The fifth will deal with the whole great subject of Diseases of the Alimentary Tract. The sixth is to group Diseases of the Kidneys, those associated with Internal Secretion, those of still obscure causation, the Diseases of the Muscles, and Vasomotor and Trophic Disorders. The seventh and final volume completes the entire subject by covering Nervous and Mental Diseases. The convenience of this grouping is manifest.

That an authoritative work presenting the whole field of medicine in its advanced state of development is required by the progress of recent years is a proposition that can not be gainsaid. To question it implies that the questioner has not kept in touch with the radical changes, the more elevated viewpoints, the more accurate methods and the improvements in details of practice which have combined to extend the scope of medicine and make it an exact and successful science instead of an empirical and uncertain art. Such an era of progress is with us now, and every practitioner desirous of maintaining his position and doing his duty to his patients must needs take cognizance or drop astern. It is fortunate for those actively engaged in practice that they can so readily gain this new knowledge combined and fitted in with what has been inherited from the past and has survived the ordeal of modern re-examination. This service is being performed for them in this work under ideal auspices, for no editor could have been chosen with a broader view of medicine in all its bearings than is possessed by Dr. Osler, nor with a keener knowledge of the best man to call upon for each constituent section. The phenomenal sale argues wide appreciation of the advantages of possessing a complete library and reference work presenting the net medicine of the new era, disembarrassed of outworn ideas, and covering the whole subject with the highest authority and practicality.

---

PROGRESSIVE MEDICINE—A Quarterly Digest of Advances, Discoveries and Improvements in the Medical and Surgical Sciences. Edited by Hobart Amory Hare, M. D., Professor of Therapeutics and Materia Medica, Jefferson Medical College, Philadelphia. Assisted by H. R. M. Landis, M. D., Asisstant Professor to Outdoor Department, Jefferson Medical College. Lea Brothers & Company, Philadelphia and New York. Six dollars per annum, or $1.50 per volume.

Volume 4, December, 1907.—Diseases of the Digestive Tract and Allied Organs, the Liver and Pancreas; Diseases of the Kidneys; Surgery of the Extremities, Fractures, Dislocations, Tumors, Surgery of Joints, Shock Anesthesia, and Infections; Genito-Urinary Diseases; Practical Therapeutics Referendum.

The contributors are Belfield, W. T. Bloodgood, J. C. Bradford, J. R. Landis, H. R. M., and Steele, J. D.

